# Emerging Role of Eosinophils in Resolution of Arthritis

**DOI:** 10.3389/fimmu.2021.764825

**Published:** 2021-10-18

**Authors:** Yi Qin, Hui-Zhi Jin, Yu-Jing Li, Zhu Chen

**Affiliations:** ^1^ Department of Rheumatology and Immunology, The First Affiliated Hospital of University of Science and Technology of China, Division of Life Sciences and Medicine, University of Science and Technology of China, Hefei, China; ^2^ Second Clinical Medical School, Second Affiliated Hospital of Fujian Medical University, Fujian Medical University, Quanzhou, China

**Keywords:** eosinophil, rheumatoid arthritis, resolution, innate lymphoid cells, alternatively activated macrophages

## Abstract

Eosinophils are a minor component of circulating granulocytes, which are classically viewed as end-stage effector cells in host defense against helminth infection and promoting allergic responses. However, a growing body of evidence has emerged showing that eosinophils are versatile leukocytes acting as an orchestrator in the resolution of inflammation. Rheumatoid arthritis (RA) is the most common chronic inflammatory disease characterized by persistent synovitis that hardly resolves spontaneously. Noteworthy, a specific population of eosinophils, that is, regulatory eosinophils (rEos), was identified in the synovium of RA patients, especially in disease remission. Mechanistically, the rEos in the synovium display a unique pro-resolving signature that is distinct from their counterpart in the lung. Herein, we summarize the latest understanding of eosinophils and their emerging role in promoting the resolution of arthritis. This knowledge is crucial to the design of new approaches to rebalancing immune homeostasis in RA, considering that current therapies are centered on inhibiting pro-inflammatory cytokines and mediators rather than fostering the resolution of inflammation.

## Introduction

Eosinophils are leukocytes that normally amount to less than 5% of white blood cells in the peripheral blood. In certain pathological settings, eosinophils significantly expand and count over 1,500 cells/μl blood, which is defined as hypereosinophilia (1). Although previously considered as the end-stage effector cells involved in helminth infection and allergic diseases like asthma, increasing evidence shows that eosinophils are multifunctional granulocytes involved in regulating adaptive immune responses, especially in inflammatory and autoimmune disorders (2).

Rheumatoid arthritis (RA) is the most common chronic immune-mediated inflammatory disease characterized by persistent synovitis that lacks self-remission (3). Although the pathogenesis of RA remains incompletely understood, the general consensus is that self-tolerance breakdown triggers autoantibody production in genetically predisposed individuals before progressing into clinically apparent RA (3). During the transition from asymptomatic autoimmunity to synovial inflammation, a diverse range of pro-inflammatory cytokines produced by immune cells such as CD4^+^ T cells, macrophages, and fibroblast-like synoviocytes emerge quickly, which eventually contribute to cartilage damage and bone erosion in the joint (4). Notably, once the joint inflammation is established, it tends to be chronic, as evidenced by the insufficiency of regulatory factors that counteract or rebalance aberrant immune responses (4). Hence, ineffective resolution of RA remains a major clinical challenge, although novel anti-inflammatory biological agents have been increasingly introduced (5, 6).

In contrast with the pro-inflammatory properties of eosinophils in asthma that cause structural remodeling of the airways, recent studies by us and collaborators have suggested that, as a crucial component of Th2 immune responses, eosinophils have previously undifferentiated pro-resolving signature in RA (7–10). In this review, the emerging role of eosinophils in promoting the resolution of arthritis is summarized. The potential underlying mechanisms that allow eosinophils to exert anti-inflammatory properties and therapeutic implications of eosinophils in arthritis are also discussed.

## Eosinophil Development and Biology

Eosinophils are generated in the bone marrow from multipotent hematopoietic stem cells, which give rise to eosinophil-committed progenitors (EoPs). These will eventually differentiate into mature eosinophils in response to several cytokines such as IL-5, IL-3, granulocyte-macrophage colony-stimulating factor (GM-CSF), and IL-33 (11–13). Eosinophilopoiesis is governed by at least three transcription factors, including GATA-1 (a zinc family finger member), PU.1 (an ETS family member), and C/EBP members (CCAAT/enhancer-binding protein family) (14, 15). Notably, GATA-1 is essential for eosinophil differentiation, since the deletion of a GATA-binding enhancer site in the GATA-1 gene generated a specific eosinophil-deficient ΔdblGATA mouse with no influence on other cell lineages (16). In addition, some microRNAs and long non-coding RNAs have been reported to be involved in eosinophilopoiesis (17–19).

Once eosinophils mature in the bone marrow, they are released into circulation and migrate into peripheral tissues under stimulation of IL-5 and eotaxin-1 (CCL11) (2). In homeostatic conditions, eosinophils are distributed in the spleen, gastrointestinal tract, thymus, adipose tissue, and uterus, indicating that they are likely to be responsible for maintaining homeostasis in different tissues (20). It is well established that eosinophils synthesize a broad range of mediators stored in granules throughout the cytoplasm, including cytotoxic granule proteins such as major basic protein (MBP), eosinophil cationic protein (ECP), eosinophil peroxidase (EPX), and eosinophil-derived neurotoxin (EDN) (21). When encountering the stimulus present in the tissue, eosinophils release granule contents rapidly, which is termed degranulation, to exert host immune defense against pathogens (2). Hence, historically it is considered that the primary effector function of eosinophils was in anti-pathogen responses, especially those involving parasites. Nonetheless, eosinophil granules also contain numerous cytokines, particularly type 2 cytokines, as well as growth factors and resolvins, suggesting their ability to be involved in tissue repair and a wide range of immunological disorders such as allergy and asthma (22, 23).

## Regulation of Th2 Responses by Eosinophils

Although they represent a minor component of innate immune cells, eosinophils are well known to be an important innate immune regulator in pathogen clearance by releasing cytokines and chemokines or by interacting with other innate immune cells (24–28). Meanwhile, increasing evidence has extended understanding that eosinophils are versatile leukocytes capable of modulating adaptive immune responses as well. For example, murine eosinophils can present antigen *via* MHC class II and promote IL-4, IL-5, and IL-13 production from antigen-specific CD4^+^ T cells in the context of helminth infection or asthma (23, 29, 30).

Other than behaving as antigen-presenting cells, eosinophils are thought to regulate Th2 immune responses in multiple ways. A Notch ligand Jagged1, which constitutes an instructive signal for Th2 differentiation, has been found to express on human eosinophils constitutively, indicating the capability of eosinophils to provide a polarization signal to naïve CD4^+^ T cells (31, 32). Studies on helminth infection models revealed that eosinophils precede lymphocyte recruitment into inflammatory sites (33, 34). In eosinophil-deficient ΔdblGATA mice infected with *Trichinella spiralis*, infiltration of Th2 cells into the muscles was highly decreased (35). In another study using IL-5/eotaxin double-knockout mice in which eosinophil counts are severely reduced, significantly decreased IL-13 production by Th2 cells in response to the OVA challenge was observed (36). Notably, this defect can be rescued by the adoptive transfer of eosinophils, suggesting the role of eosinophils in the regulation of Th2 immune responses (36). In addition to the secretion of Th2-related cytokines, eosinophils can also promote Th2 responses through the synthesis of indoleamine 2,3-dioxygenase (IDO), an enzyme that catalyzes the oxidative catabolism of tryptophan to kynurenines (37).

## Eosinophils Promote the Resolution of Inflammation

Inflammation is an evolutionary defensive host response to injury, characterized by the recruitment of leukocytes and cytokines from the circulation to the inflamed tissue. Generally, acute inflammation in healthy individuals is self-limited and resolves timely, thus preventing to progress to chronic inflammation (38). During the course of acute inflammation, the migration of polymorphonuclear neutrophils (PMNs) into tissues is the early event, followed by the recruitment of monocytes that will further differentiate into tissue macrophages. It is known that a variety of classic proinflammatory mediators such as prostaglandins (PGs) and leukotrienes (LTs) coordinate these initial events of acute inflammation by regulating vascular permeability and leukocyte infiltration (39). Once the malicious components are removed by phagocytosis, the inflammatory response must be promptly resolved to prevent excessive tissue damage and return to homeostasis. However, uncontrolled or long-lasting inflammation is believed to exist in the pathogenesis of many human autoimmune and inflammatory diseases including RA (40).

In recent years, a growing body of evidence has emerged that shows that resolution of acute inflammation is not a passive but an active process controlled by endogenous resolving mediators, termed specialized pro-resolving mediators (SPMs) such as protectins, resolvins, and maresins, which belong to families of lipid mediators (41). Blocking lipid mediator biosynthesis by either cyclooxygenase (COX)-2 or lipoxygenase (LOX) inhibitors resulted in resolution defect, which is characterized by sustained leukocyte infiltration in inflamed sites and impaired removal of phagocytes to the draining lymph nodes (42), suggesting the critical role of these lipid mediators in regulating the timely resolution of acute inflammation.

Interestingly, eosinophils are an orchestrator in the resolution of inflammation. In a murine zymosan-induced peritonitis model, eosinophils were recruited to the inflamed site during the resolution phase of acute peritonitis (43). Liquid chromatography–tandem mass spectrometry (LC-MS/MS)-based lipidomics analyses revealed that pro-resolving mediators such as protectin D1 (PD1) were increased during the resolution phase in a 12/15-LOX dependent manner (43). PD1 promotes the resolution process by inhibiting PMN influx and stimulating macrophage ingestion of apoptotic PMNs, as well as increasing phagocyte clearance into draining lymph nodes (42). Importantly, the researchers revealed that eosinophils were the main PD1-producing cells in the resolution phase of zymosan-induced peritonitis (43). Depletion of eosinophils or CXCL13 *in vivo* caused a resolution defect, characterized by impaired lymphatic drainage of inflammatory phagocytes carrying engulfed zymosan in the draining lymph node, and delayed removal of PMNs in the inflamed tissues (44). Notably, administration of PD1, CXCL13, or adoptive transfer of eosinophils from wild-type but not from 12/15-LOX deficient mice reversed the defective phenotype of the resolution process, suggesting that eosinophils promote the resolution of inflammation through pro-resolving mediators and CXCL13 pathway (43, 44). In another experimental colitis model, more severe colitis was observed in eosinophil-deficient mice compared with wild-type controls, accompanied with decreased level of PD1 in the colon (45). Furthermore, administration of exogenous PD1 alleviated the severity of colitis and reduced neutrophil infiltration. All these findings indicated that eosinophils contribute to the resolution of inflammation by producing pro-resolving lipid mediators such as PD1 *via* the 12/15-LOX-mediated biosynthetic pathway.

## Regulatory Eosinophils in RA

Although eosinophils act as a counter regulator in several inflammatory disorders, the association between eosinophils and the development of RA was largely undetermined, probably due to the uncommon clinical manifestation of eosinophilia in RA. Indirect evidence from previous studies reported that RA patients, especially those with high activity and short disease duration, have increased serum levels of ECP, supporting the notion that eosinophils were involved in the inflammatory responses of RA (46, 47). In a prospective multi-center cohort study, the prevalence of eosinophilia in patients with new-onset arthritis (disease duration ranges from 6 weeks to 6 months) is only 3.2% (48). Notably, after 3 years, patients with eosinophilia presented with signs of higher disease activity compared with those without eosinophilia at baseline, suggesting that baseline eosinophilia might be a poor prognosis marker in early arthritis patients (48). A more recent prospective observational study investigated clinical characteristics of RA patients with persistent eosinophilia and compared with the patients without eosinophilia (49). After excluding secondary causes of eosinophilia such as concomitant allergic diseases and intestinal helminth infection, the authors did not find differences in clinical features between RA patients with or without eosinophilia (49). The discrepancies among studies might reflect the complex heterogeneity of eosinophil phenotype or function in inflammatory arthritis.

Recently, we have shown that the expression of synovium EPX was elevated in RA patients compared with osteoarthritis (OA) patients (7). Consistently, serum EPX level was higher in RA patients than pre-RA and healthy controls (7). In the K/BxN serum-induced arthritis model, IL-5 transgenic (IL-5tg) mice that have extraordinary hypereosinophilia showed a significant reduction of arthritis score, whereas eosinophil-deficient ΔdblGATA mice presented with higher disease activity (7). In addition, the adoptive transfer of eosinophils into collagen-induced arthritic mice led to the alleviation of arthritis, accompanied by reduced joint inflammation and bone erosion in histology evaluation (9). Altogether, these data suggested that eosinophils have previous unknown pro-resolving properties in promoting the resolution of inflammatory arthritis.

Considering the dual nature of eosinophils as pro-inflammatory and pro-resolving cells, it is reasonable to speculate that eosinophils have different subsets responsible for different biological functions. Indeed, this is supported by the research that revealed two distinct eosinophil subsets, lung resident eosinophils and recruited inflammatory eosinophils, in asthmatic lungs (50). Remarkably, a very recent study described a specific population of eosinophils, named regulatory eosinophils (rEos), was present in the joints of arthritic mice as well as synovium of RA patients (8). OVA allergen challenge triggered an earlier resolution of K/BxN serum-induced arthritis, accompanied with increased eosinophils in the arthritic joints. Strikingly, this protective manifestation was only observed in wild-type but not eosinophil-deficient ΔdblGATA mice, indicating the essential role of eosinophils in the asthma-induced resolution of joint inflammation (8). In further analyses, both single-cell RNA sequencing and proteome profile analyses confirmed that the rEos in the joint display a unique pro-resolving characteristic, which is distinct from their counterpart in the lung. For example, joint rEos have strongly upregulated expression of 5-LOX and 12/15-LOX (8), which could explain the anti-inflammatory and pro-resolving effector function of rEos, since the deletion of 12/15-LOX was linked to uncontrolled inflammation and tissue damage in chronic arthritis (51). Moreover, rEos has a specific secretion pattern compared with its inflammatory counterpart in the lung, characterized by the production of MMP-3, osteopontin, and serpin E1, suggesting that rEos might also foster synovial tissue recovery besides ceasing inflammation (8). Interestingly, rEos was infiltrated more frequently in RA patients in remission than patients in the active stage. Inactive RA patients with concomitant asthma developed a flare of disease after anti-IL-5 monoclonal antibody treatment, which might be explained reasonably by the depletion of rEos (8).

## ILC2–Eosinophil–M2 Macrophage Axis Counteracts Joint Inflammation

As a messenger between the innate and adaptive immune systems, innate lymphoid cells (ILCs) have been realized to be intensely involved in the pathogenesis of inflammatory arthritis (52, 53). In particular, ILC2 acts as a crucial regulator in damping joint inflammation in contrast to proinflammatory ILC1/ILC3. Circulating ILC2 count was inversely correlated with disease activity index in RA patients and increased after receiving anti-rheumatic treatment (54). In line with these human data, genetic deletion of ILC2 in mice aggravated K/BxN serum-induced arthritis whereas expansion of ILC2 by IL-25/IL-33 mini-circles or adoptive transfer of ILC2 from wild-type but not IL-4^-/-^IL-13^-/-^ mice attenuated arthritis (55). Furthermore, ILC2 was found to inhibit osteoclast differentiation and bone loss independently of inflammation (56).

Interestingly, it has been established that tissue-resident ILC2 regulates eosinophil homeostasis and accumulation into tissues through constitutive secretion of IL-5 (57). In asthmatic lung, ILC2 was the main producer of IL-5, which consequently drives the expansion and infiltration of rEos into the arthritic joints (8). Another recent work by us showed that activation of ILC2 by a small neuropeptide significantly suppressed the development of collagen-induced arthritis, accompanied by the expansion of eosinophils in the arthritic joints (10). In addition, induction of ILC2 by administration of IL-25/IL-33 accelerated the resolution of K/BxN serum-induced arthritis in wild-type but not eosinophil-deficient ΔdblGATA mice (8). On the contrary, neutralization of IL-5 by a monoclonal antibody blocked asthma-induced resolution of arthritis, with a reduced expansion of rEos in the joints (8). All these results supported the perspective that eosinophils are indispensable for ILC2-mediated resolution of arthritis (8).

**Figure 1:**
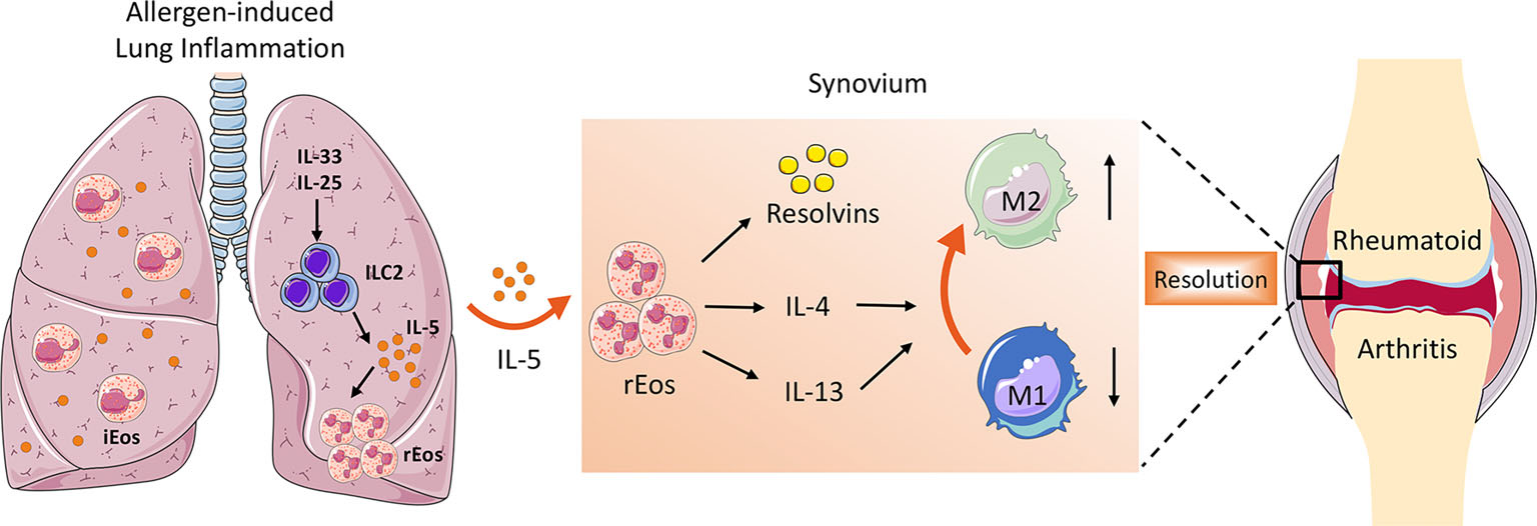
The role of ILC2–eosinophil–M2 macrophage axis in promoting resolution of arthritis. In allergen-triggered lung inflammation, ILC2 was expanded and activated in response to stimulators such as IL-33 and IL-25. Systemically elevated IL-5 secreted by ILC2 drives proliferation and recruitment of rEos into the inflamed joints, where they produce a variety of lipid mediators and anti-inflammatory type 2 cytokines, which facilitate M2 macrophage priming and eventually contribute to the resolution of arthritis.

Besides secreting a variety of pro-resolving lipid mediators that are crucial for the resolution of inflammation, the role of eosinophils in the suppression of arthritis could also include switching macrophages from pro-inflammatory M1 to anti-inflammatory M2 phenotype. As is well known, synovial macrophages act as central effector cells in the development of synovitis (3). The abundant presence of pro-inflammatory cytokines such as TNFα, IL-6, and IL-1β in the inflamed synovium suggests a predominant M1 macrophage phenotype in RA. However, the phenotype of synovial macrophages *in vivo* is highly complex and often exhibit a mixed polarization state (58). Previously it has been reported that even the same M1 macrophages recruited during the initial phase of arthritis can switch their phenotype toward M2 macrophages (59). In contrast to M1 macrophages that facilitate the inflammatory cascade in the synovium, M2 macrophages halt joint inflammation by removing dead cells (efferocytosis) and producing pro-resolving lipid mediators (58). It has been demonstrated that eosinophils induce polarization of macrophages toward M2 phenotype through secretion of IL-4, IL-13, and 12/15-LOX-derived lipid mediators (43). Eosinophil deficiency was associated with the impaired distribution of anti-inflammatory MHC-II^-^ macrophages in the steady state as well as in arthritis (7). Both *in vitro* and *in vivo* studies showed that eosinophils foster the polarization of M1 to M2 macrophages in the synovial tissue, partly *via* the IκB/P38 MAPK signaling pathway (8, 9). This is consistent with previous studies that reported that eosinophils in adipose tissue mediate macrophage differentiation into M2 phenotype, which are required for glucose homeostasis (60, 61). Taken together, the ILC2–eosinophil–M2 macrophage axis represents a novel and important immunological pathway counteracting joint inflammation and eliciting resolution of arthritis (**Figure 1**).

## Future Perspective in Therapy

With accumulating evidence and advancing technologies, the classical view of eosinophils has changed from a pro-inflammatory cell in helminth infection and allergy to a cell type aggressively involved in anti-inflammatory responses in the resolution of chronic inflammation. The existence of rEos in the synovium of RA patients extended previous understanding that eosinophils are critical in counteracting joint inflammation and facilitating the resolution of disease. These findings are crucial for designing new approaches to rebalancing immune homeostasis in inflammatory arthritis, considering that current therapies are centered on inhibiting pro-inflammatory cytokines and mediators rather than fostering the resolution of inflammation. Hence, understanding how rEos are activated and expanded will offer a novel strategy for the development of safe and effective treatment for arthritis.

Indeed, it is well known that the major extrinsic driver of eosinophil expansion was helminth infection. Numerous previous studies in experimental mouse models have demonstrated clinical improvement of inflammatory activity in a variety of autoimmune and inflammatory diseases including RA (7, 62–67). These observations led to the proposal that harness helminth and their secreted products would represent a promising interventional approach for the treatment of RA, as supported by the findings that a filarial nematode-derived glycoprotein, ES-62, has been proved to exert an anti-inflammatory and anti-osteoclastogenic effect in mouse arthritis models (68–71). In addition, several clinical trials have been performed to evaluate the immune-regulatory effect of helminth on autoimmune diseases, especially in inflammatory bowel disease (72). The mechanisms by which helminth and their derivatives modulate the host’s immune system were attributed to shifting immune responses from Th1 to Th2, induction of regulatory T and B cell subsets, as well as downregulation of IFN-γ and IL-17 (73). This helminth-based immunotherapy is becoming of major interest since current conventional management of RA relies generally on nonspecific inhibition of the immune system, which often results in severe infections and malignancies. Consistent with this concept, one of our recent studies showed that a small neuropeptide named Neuromedin U successfully alleviated collagen-induced arthritis, with evidence of ILC2-eosinophil activation (10). On the other hand, the plasticity of eosinophils offers another strategy that induces differentiation of pro-inflammatory eosinophils into regulatory phenotypes.

## Conclusion

In summary, emerging evidence has shown that eosinophils not only act as a pro-inflammatory effector cell but also display a pro-resolving feature in RA. They consistently reside in the synovium of RA patients in remission and proliferate under stimulation of ILC2-derived IL-5. Mechanistically, the rEos promote the resolution of arthritis through secreting resolvins in a 12/15-LOX-dependent manner and switching synovial macrophages into the M2 phenotype.

## Author Contributions

YQ, H-ZJ, and Y-JL drafted the manuscript. ZC revised the manuscript. All authors contributed to the discussion and approved the submitted version.

## Funding

This work was supported by the National Natural Science Foundation of China (Grant Nos. 81871227 and 81501344).

## Conflict of Interest

The authors declare that the research was conducted in the absence of any commercial or financial relationships that could be construed as a potential conflict of interest.

## Publisher’s Note

All claims expressed in this article are solely those of the authors and do not necessarily represent those of their affiliated organizations, or those of the publisher, the editors and the reviewers. Any product that may be evaluated in this article, or claim that may be made by its manufacturer, is not guaranteed or endorsed by the publisher.
